# Preoperative Impella therapy in patients with ventricular septal rupture and cardiogenic shock: haemodynamic and organ function outcomes

**DOI:** 10.1093/icvts/ivae137

**Published:** 2024-07-23

**Authors:** Ikuko Shibasaki, Shunsuke Saito, Yuta Kanazawa, Yusuke Takei, Go Tsuchiya, Hirotsugu Fukuda

**Affiliations:** Department of Cardiac and Vascular Surgery, Dokkyo Medical University School of Medicine, Tochigi, Japan; Department of Cardiac and Vascular Surgery, Dokkyo Medical University School of Medicine, Tochigi, Japan; Department of Cardiac and Vascular Surgery, Dokkyo Medical University School of Medicine, Tochigi, Japan; Department of Cardiac and Vascular Surgery, Dokkyo Medical University School of Medicine, Tochigi, Japan; Department of Cardiac and Vascular Surgery, Dokkyo Medical University School of Medicine, Tochigi, Japan; Department of Cardiac and Vascular Surgery, Dokkyo Medical University School of Medicine, Tochigi, Japan

**Keywords:** Cardiogenic shock, Postinfarction ventricular septal rupture, Impella, Venoarterial extracorporeal membrane oxygenation, Myocardial change

## Abstract

**OBJECTIVES:**

We examined the effects of preoperative Impella treatment on haemodynamic stability, organ recovery and postoperative outcomes in patients with postinfarction ventricular septal rupture (PIVSR) and cardiogenic shock (CS).

**METHODS:**

Between April 2018 and February 2024, the data of 10 of 15 patients with PIVSR and CS who underwent Impella therapy were analysed. Emergency surgery was contingent on haemodynamic stability with the Impella/ECpella, except in the presence of organ failure. We utilized a generalized linear mixed model to evaluate organ ischaemia through changes in blood parameters upon admission and at subsequent intervals post-Impella insertion.

**RESULTS:**

Preoperative Impella or combined Impella and ECpella (5 patients each) support was provided, with diagnoses and operations occurring at an average of 4 days (interquartile range: 2–5) and 8 days (interquartile range: 2–14) after myocardial infarction, respectively. Treatment significantly reduced lactate, alanine aminotransferase, creatine kinase-MB and troponin I levels (*P* ≤ 0.05 for all). Conversely, no significant change was noted in the aspartate aminotransferase level or the estimated glomerular filtration rate. Haemoglobin and platelet counts decreased despite transfusions (*P* < 0.001). No surgical deaths occurred; however, 70% of the patients required prolonged mechanical ventilation, and 80% were transferred to other facilities for rehabilitation.

**CONCLUSIONS:**

Impella or ECpella treatment can improve haemodynamic and organ failure outcomes in patients with PIVSR and CS. However, the risks of prolonged support, including haemorrhagic events and the need for extended rehabilitation, point to a need for comparative studies to optimize support duration.

## Graphical Abstract

**Figure:**
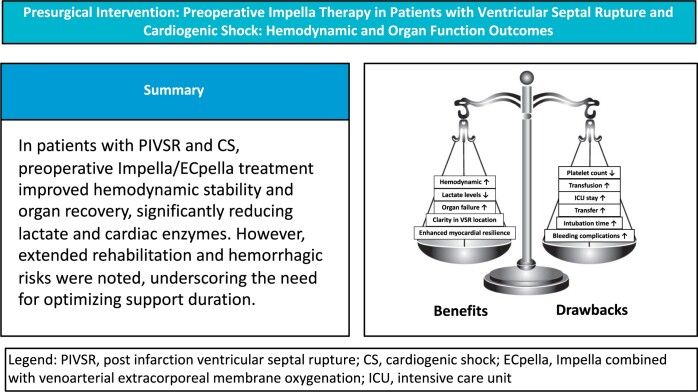

## INTRODUCTION

Despite recent advancements in early revascularization strategies that have reduced the incidence of postinfarction ventricular septal rupture (PIVSR) to 0.2%, PIVSR remains a lethal complication [1, 2]. Emerging approaches in interventional techniques have drawn attention to the percutaneous closure of PIVSR, with the optimal timing for closure reported as 3 weeks post-acute myocardial infarction (AMI) [3]. However, comparisons between surgical and percutaneous treatments have shown that surgical intervention significantly reduces in-hospital deaths and the incidence of residual shunts, while also improving postoperative cardiac function. Thus, these findings confirm the preference of surgical repair as an appropriate treatment method [4].

Patients with PIVSR experience both left heart failure (HF) due to AMI and right HF due to the VSR shunt; thus, early surgical intervention is considered beneficial. However, the acute myocardial damage-related vulnerability of the myocardium surrounding the PIVSR and organ dysfunction induced by cardiogenic shock (CS) complicate early repair. The optimal timing of surgery for PIVSR remains controversial. Current recommendations suggest proceeding with early surgical repair if the patient’s condition deteriorates after mechanical circulatory support (MCS). Alternatively, the operation may be delayed for 3–4 weeks if MCS stabilizes the haemodynamics [5].

Recently, the Impella device (Abiomed, Danvers, MA, USA) was introduced in Japan and has been used as a bridging support to surgery for patients with PIVSR. After insertion of the Impella device, there was a significant reduction in the Qp/Qs shunt ratio, systolic pulmonary artery pressure and pulmonary capillary wedge pressure, along with an effective increase in the cardiac index (CI) [6]. Furthermore, the combination of the Impella and veno-arterial extracorporeal membrane oxygenation (VA-ECMO), known as ECpella, has significantly improved outcomes in patients with severe acute coronary syndrome complicated by CS, compared with the combined treatment of intra-aortic balloon pump (IABP) and VA-ECMO [7].

Although treatment strategies using MCS as a bridge to a VSR closure operation have attracted considerable attention, preoperative data regarding these strategies for PIVSR are limited to case reports and small case series. The goal of this study was to examine the haemodynamic and organ effects of Impella treatment as preoperative management for patients with PIVSR accompanied by CS as well as its impact on postoperative outcomes.

## PATIENTS AND METHODS

### Ethics statement

The study was approved by the Dokkyo Medical University Hospital Ethics Committee (approval No: R-47-9J) on 20 August 2020. This approval covered both prospective and retrospective aspects of the study. Written informed consent was obtained from all prospective participants. For the retrospective analysis, the ethics committee waived the requirement for individual consent.

### Patients and therapeutic strategy

**Figure 1:**
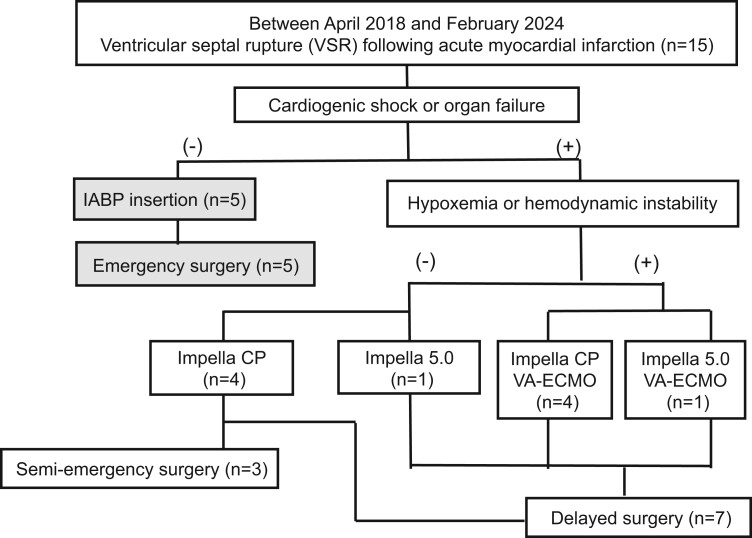
Study flow chart. IAPB: intra-aortic balloon pump; VA-ECMO: veno-arterial extracorporeal membrane oxygenation.

Patients with PIVSR from April 2018 to February 2024 at our institute who underwent surgical repair, experienced CS and had preoperative Impella support were included in the present study; those without Impella support were excluded (Fig. 1). Consent was obtained unanimously without refusal.

The primary end points were laboratory trends and complications during Impella management and perioperative complications and deaths. The secondary outcomes were myocardial changes from Impella use.

Our PIVSR management strategy involves the initial use of an IABP and emergency surgery if CS or organ failure is absent at diagnosis. In patients with CS, we use Impella support, which is transitioned to ECpella (addition of VA-ECMO) if hypoxaemia or haemodynamic instability occurs; the operation is then postponed until organ recovery, with the timing determined by the surgeon. Upgrading to ECpella is required if haemodynamic stability is compromised, whereas uncontrollable haemorrhagic complications or worsening organ failure necessitates semi-emergency surgery.

### Definitions

CS was diagnosed using the following criteria: systolic blood pressure < 90 mmHg for over 30 min or vasopressor requirements to achieve higher blood pressure; signs of pulmonary congestion or elevated left-ventricular pressure; and symptoms of organ perfusion impairment, such as altered mental status, cold skin, oliguria or high lactate levels [8]. The day of Impella insertion was counted as day 0, with the subsequent day as day 1. Protocols for administering red cell concentrates (RCC) and platelet concentrates (PC) were based on haemoglobin (Hb) levels < 10 g/dl and platelet counts < 30 × 10^3^/µl, at the discretion of the clinician.

### Data collection

Patient records were used to gather demographic, anatomical, surgical and perioperative data. During Impella support, daily blood samples and images from chest radiography and transthoracic echocardiography were analysed. Organ ischaemia improvement was monitored via clinical laboratory including aspartate aminotransferase, alanine aminotransferase and lactate levels and the estimated glomerular filtration rate. Myocardial damage was assessed using creatine kinase (CK)-MB and troponin levels, whereas haemorrhagic complications were evaluated through lactate dehydrogenase and Hb levels, platelet count and transfusion volumes of RCC, fresh-frozen plasma and PC.

### Statistical analyses

**Figure 2:**
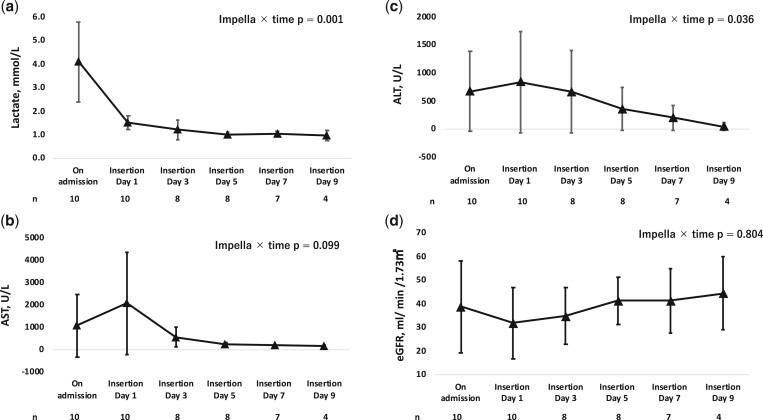
Efficacy of Impella treatment on (**a**) lactate levels, (**b**) aspartate aminotransferase levels, (**c**) alanine aminotransferase levels and (**d**) estimated glomerular filtration rate over time. Changes in blood data from admission to 1, 3, 5, 7 and 9 days after Impella insertion were analysed using a generalized linear mixed model. ALT: alanine aminotransferase; AST: aspartate aminotransferase; eGFR: estimated glomerulofiltration rate.

**Figure 3:**
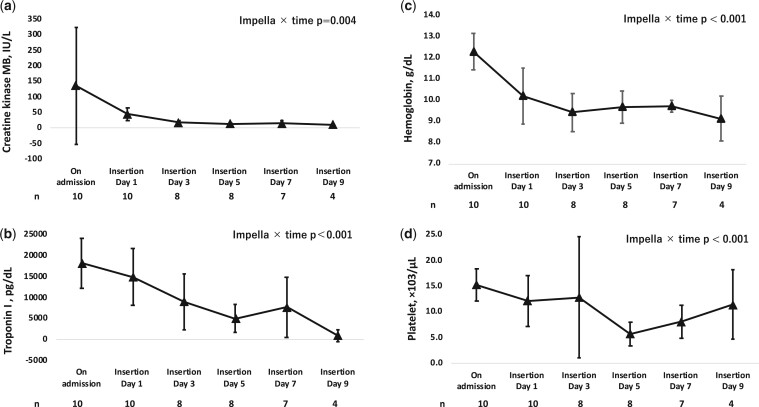
Efficacy of Impella treatment on the levels of (**a**) creatine kinase-MB, (**b**) troponin I, (**c**) haemoglobin and (**d**) platelets over time. Changes in blood data from admission to 1, 3, 5, 7 and 9 days after Impella insertion were analysed using a generalized linear mixed model.

Due to the limited number of cases, continuous variables are presented as medians with interquartile ranges (IQR), and categorical data are summarized using frequency tables. Given the small sample size, for the total CI for Impella and ECpella, we used the mean (standard deviation). In this study, the median duration of Impella support was 9 days post-insertion. To analyse changes in the haematological data from admission to days 1, 3, 5, 7 and 9 after Impella insertion, a generalized linear mixed model was used (Figs 2a–d and 3a–d). A complete case analysis was used to address missing data, analysing only cases with fully available data. Although this method may introduce biases by excluding incomplete cases [9], it was appropriate given our study’s context and the missing data patterns. The number of cases at each time point, excluding missing values, was reported to ensure transparency and facilitate evaluation of the integrity of the data. All statistical analyses were conducted using SPSS Statistics 29.0 (IBM SPSS, Inc, Armonk, NY, USA).

## RESULTS

### Characteristics

The study flow chart of patients who underwent PIVSR surgical repair at Dokkyo Medical University Hospital is shown in Fig. 1. The present study included 10 preoperatively Impella-supported patients; their baseline characteristics are detailed in Table 1. The median age was 74.5 years (IQR: 69.5–78.5), and 70% were male. The interval from AMI onset to PIVSR diagnosis was 4 days (IQR: 2–4) and that from diagnosis to surgery was 8 days (IQR: 2–14). Among the 10 patients, 5 additionally received VA-ECMO due to hypoxaemia or haemodynamic instability. Additionally, 8 were approached via the femoral artery with Impella CP, from which 6 were intubated. Two patients with Impella 5.0 had axillary insertions and were intubated, 1 with ECpella and the other with Impella alone, with the latter requiring on-bed rehabilitation due to the inability to extubate. For all 10 patients, no cases of right-to-left shunts developed during Impella support.

**Table 1:** Baseline clinical characteristics and preoperative events (*n* = 10)

Variables	Overall, *n* (%) or median (IQR)
Age (years)	74.5 (69.5–78.5)
Male sex	7 (70.0%)
Body mass index (kg/m^2^)	20.9 (20.2–22.3)
Hypertension	5 (50.0%)
Dyslipidaemia	4 (40.0%)
Diabetes mellitus	2 (25.0%)
Smoking	3 (30.0%)
Culprit vessel	
LAD	9 (90.0%)
RCA	1 (10.0%)
AMI to VSR (days)	4 (2–5)
VSR to surgery (days)	8 (2–14)
PCI (DES) procedure	3 (30.0%)
Impella CP	8 (80.0%)
Impella 5.0	2 (20.0%)
ECpella	5 (50.0%)
Intubation	8 (80.0%)
Impella treatment duration (days)	9 (1–14)
VA-ECMO treatment duration (days)	5 (4–10)
Total CI (L/min/m^2^)	
Insertion day 1 (*n* = 10; ECpella *n* = 5)^a^	2.6 ± 0.9 (VA-ECMO CI: 1.9 ± 0.8)
Insertion day 3 (*n* = 7; ECpella *n* = 5)^a^	3.1 ± 0.9 (VA-ECMO CI: 2.0 ± 0.5)
Insertion day 5 (*n* = 7; ECpella *n* = 4)^a^	2.6 ± 0.6 (VA-ECMO CI: 2.2 ± 0.3)
Insertion day 7 (*n* = 6; ECpella *n* = 3)^a^	2.5 ± 0.5 (VA-ECMO CI: 2.0 ± 0.1)
Insertion day 9 (*n* = 4; ECpella *n* = 1)^a^	2.4 ± 0.5 (VA-ECMO CI: 1.9)
Complication (preoperative)	
Device-related bleeding	7 (70.0%)
Nosebleed	7 (70.0%)
Gastrointestinal bleeding	3 (30.0%)
Haematuria	3 (30.0%)
Cerebral haemorrhage	2 (20.0%)
Transfusion (preoperative)	
Red blood cells (U)	18 (5–26)
Fresh-frozen plasma (U)	14 (4–20)
Platelet concentrate (U)	30 (10–48)

a Mean (SD).

AMI: acute myocardial infarction; CI: cardiac index; DES: drug-eluting stent; ECpella: Impella plus veno-arterial extracorporeal membrane oxygenation; IQR: interquartile range; LAD: left anterior descending artery; PCI: percutaneous coronary intervention; RCA: right coronary artery; VA-ECMO: veno-arterial extracorporeal membrane oxygenation; VSR: ventricular septal rupture.

### Efficacy of Impella treatment

The trends in laboratory data during mechanical support are shown in Figs. 2 and 3. Significant reductions in lactate, ALT, CK-MB and troponin levels were observed, with particularly pronounced declines in lactate and CK-MB from day 1 of Impella insertion (Figs 2a and 3a). Although an increase in estimated glomerular filtration rate was observed, the difference was not significant. Additionally, significant decreases in Hb and platelet counts were observed, despite transfusions.

### Outcomes of surgical interventions and perioperative management

Table 2 presents the outcomes of surgical interventions and perioperative management. The Daggett and double-patch methods were used for VSR repair, with concomitant coronary artery bypass grafting performed in 5 patients. At the time of weaning off cardiopulmonary bypass, 2 patients required support with an IABP alone, 1 required IABP+VA-ECMO and 1 was supported with the ECpella. Re-sternotomy was performed in approximately one-third of patients.

**Table 2:** Outcomes of surgical interventions and perioperative management (*n* = 10)

Variables	Overall, *n* (%) or median (IQR)
Surgical procedure	
Daggett	6 (60.0%)
Double patch technique	4 (40.0%)
Concomitant CABG	5 (50.0%)
Operation time (min)	346 (302–461)
CPB time (min)	162 (142–210)
Aorta cross-clamp time (min)	128 (108–154)
Mechanical circulatory support	
IABP	2 (20.0%)
IABP+VA-ECMO	1 (10.0%)
ECpella	1 (10.0%)
Transfusion	
Red-cell concentrate (U)	10 (9–16)
Fresh-frozen plasma (U)	16 (10–20)
Platelet concentrate (U)	30 (20–30)
Redo sternotomy	3 (30.0%)
Intubation time (h)	168 (72–264)
ICU stay (days)	11 (6–19)

CABG: coronary artery bypass graft; CPB: cardiopulmonary bypass; ECpella: Impella plus VA-ECMO; IABP: intra-aortic balloon pump; ICU: intensive care unit; IQR: interquartile range; VA-ECMO: veno-arterial extracorporeal membrane oxygenation.

**Figure 4:**
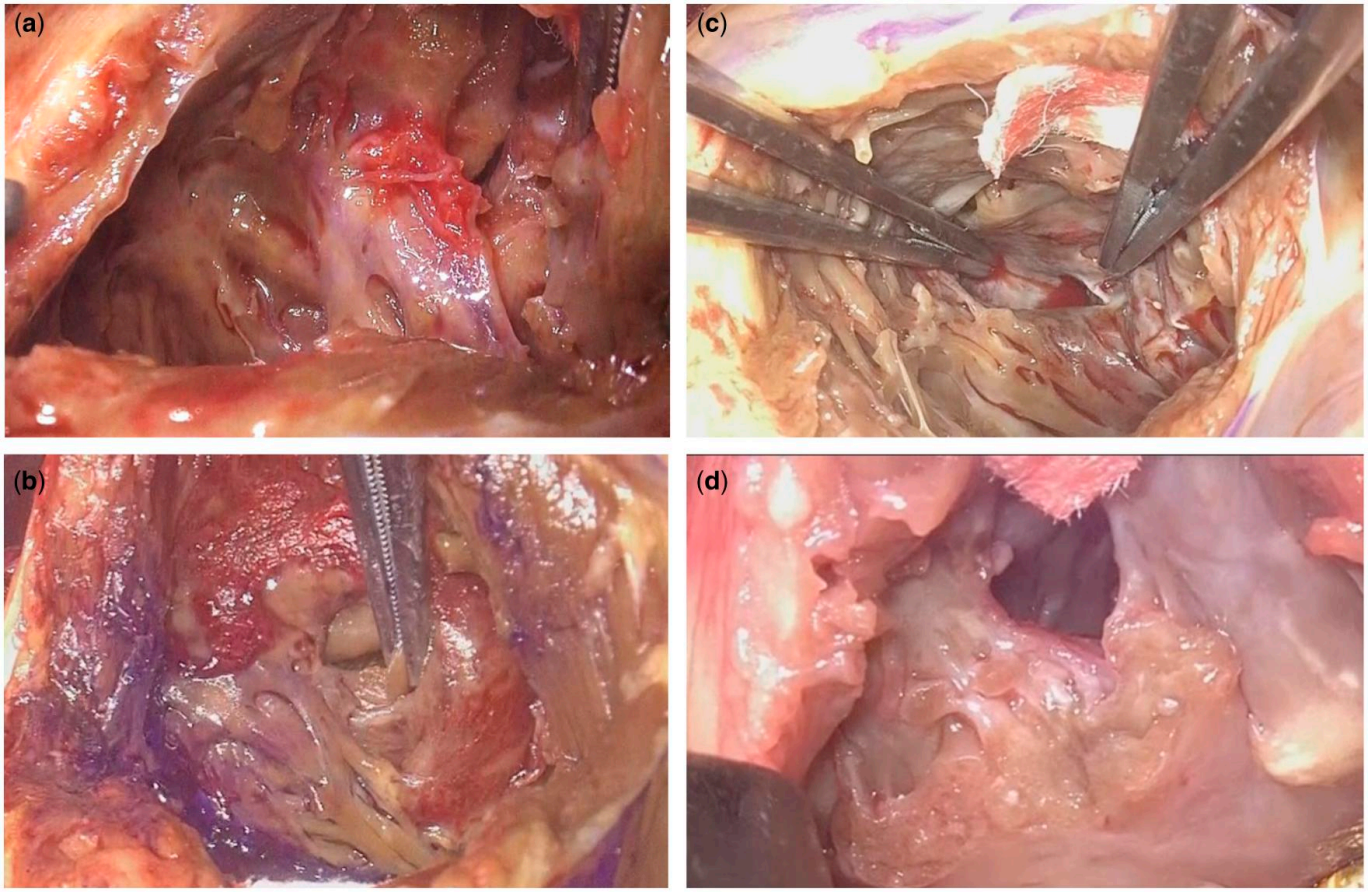
Ventricular septal rupture lesion and surrounding tissues as seen during the operation. Impella treatment duration and the time from acute myocardial infarction onset to surgery are as follows: (**a**) 1 day and 3 days, (**b**) 8 days and 11 days, (**c**) 13 days and 14 days and (**d**) 16 days and 24 days in cases a–d, respectively.

Figure 4 shows the surgical images of the VSR and surrounding tissues during surgery in 4 cases. In case a, with 2 days of Impella treatment, the VSR location was indistinct, and the surrounding tissues were fragile. In case B, with 9 days of Impella treatment, the VSR location was clear; however, the tissues remained fragile. Conversely, in cases c and d, with >14 days of Impella treatment, the VSR location was clearly identifiable, and the surrounding tissues had recovered. Interestingly, although markers such as CK-MB and troponin I showed an improving trend from the first day of Impella insertion (Fig. 3a and b), the myocardial condition was such that the location of the VSR became clear first, followed by the recovery of the surrounding tissues.

### Mortality and complications associated with Impella use for postinfarction ventricular septal rupture

There were no in-hospital deaths (cardiac or non-cardiac), and there was only 1 non-cardiac death in the mid-term results, with a follow-up period of 25 months (IQR: 16–40). The mean length of postoperative hospital stay was 66 days (IQR: 40–90). Regarding postoperative complications, prolonged intubation exceeded 72 h in 7 patients, and new-onset dialysis was initiated in 3 patients. Complications associated with MCS included cerebral infarction in 2 patients and peripheral vascular dissection in 1 patient. Additionally, 8 patients were transferred to a rehabilitation hospital due to disuse syndrome from prolonged bed rest. Of these, 7 were discharged, and 1 died of pneumonia (Table 3).

**Table 3:** Deaths and complications associated with Impella use for postoperative ventricular septal rupture (*n* = 10)

Variables	Overall, *n* (%)
Hospital transfers	8 (80.0%)
In-hospital deaths	0
Mid-term death [25 months (IQR 16–40)]	1 (10.0%)
Complications	
Intubation time >72 h	7 (70.0%)
Dialysis	4 (40.0%)
Stroke	2 (20.0%)
Tracheostomy	3 (30.0%)
Sternal osteomyelitis	1 (10.0%)
Haemorrhagic rectal ulcer	1 (10.0%)
Peripheral vascular dissection	1 (10.0%)
Reoperation	1 (10.0%)

IQR: interquartile range.

## DISCUSSION

We explored the haemodynamic and organ effects of preoperative Impella treatment in patients with PIVSR accompanied by CS and its potential impact on postoperative outcomes. The proactive deployment of the Impella as MCS appeared to help in stabilizing blood flow and mitigating organ ischaemia and permitted deferring the operation to a more clinically suitable time without resulting in any surgery-related deaths. Importantly, the mortality rate was lower than the operative mortality previously reported for patients with CS following a myocardial infarction who developed VSR [10]. Nevertheless, the observations of reduced Hb levels and platelet counts, extended intubation periods and a higher incidence of transfers to rehabilitation facilities raise concerns.

Although its prevalence has decreased, the incidence and mortality rates of PIVSR remain significant [11]. VSR typically occurs 3–8 days after AMI onset [12]; we observed an average of 4 days (IQR; 2–5) for the diagnosis of PIVSR in this study.

The European Society of Cardiology guidelines state that extracorporeal life support and VA-ECMO are used for short-term support as a ‘bridge to decision-making’ in patients with acute, rapidly worsening HF or CS. This approach helps to stabilize haemodynamics and restore end-organ function [13]. The Impella is also used as a bridge to surgery. Studies have shown that a longer duration from AMI onset to surgery in patients diagnosed with VSR correlates with lower mortality rates, whereas an emergency VSR operation in patients with CS is associated with poor outcomes [5, 14, 15]. However, delaying an operation using an MCS strategy is considered ideal for reducing mortality rates [12, 16–18].

Available support strategies include IABP, VA-ECMO and the Impella. According to Pahuja *et al.* [19], no percutaneous MCS can normalize haemodynamics in a simulator with a ventricular septal defect (VSD) size of 16.5 mm in diameter. IABP support, which reduces arterial afterload, has been observed to increase the total blood flow and decrease the number of VSD shunts. VA-ECMO support increases overall blood flow and shows an improvement trend, as well as increases VSD shunt volume and left-ventricular afterload pressure, resulting in increased pulmonary pressure. However, blood oxygenation significantly increased oxygen saturation across all areas. In contrast, support from the Impella, which pumps blood directly from the left ventricle to the aorta, reduces the pressure gradient between the left and right ventricles by lowering the pressure in the left ventricle, significantly decreasing the shunting from left to right through the VSD; moreover, it increased mean arterial pressure and decreased pulmonary capillary wedge pressure. They reported that a combination of the Impella 5.5 and VA-ECMO is the most ideal regarding haemodynamics and pressure-volume loops.

Seven authors have reported Impella use as the primary MCS strategy [6, 12, 18, 20–23], involving a total of 12 patients, with a preoperative MCS duration of 12.0 ± 4.8 days. The surgical mortality rate was 16.7%; however, apart from 1 report [6], no other deaths were reported. Similarly, no surgical deaths were observed in this study either.

The key aspects of the preoperative management of VSR include recovery from CS and stabilization of haemodynamics until surgical closure can be performed. La Torre *et al.* [6] used the Impella 5.0 in 5 patients with PIVSR and CS and administered the treatment for 14.4 ± 6 days. In their series, although 1 case required haemodialysis, the average serum creatinine levels decreased during mechanical support, and liver function improved, as indicated by decreased ALT, AST and total bilirubin levels. Before the Impella was implanted, the CK-MB values significantly exceeded 10% of the total CK, but they normalized to below 10% within 15 days after the implant was performed. Conversely, Morimura *et al.* [17] used IABP and VA-ECMO in 8 patients with PIVSR and CS, with support durations of 43.2 (range: 24.9–54.3) h and 36.9 (range: 22.0–48.1) h, respectively. In our series, 5 patients required ECpella treatment to maintain haemodynamics, achieving stabilization with an average CI of 2.6 l/min/m^2^ during MCS treatment. This approach led to significant improvements in ALT, lactate, CK-MB and troponin levels compared with those at admission, indicating a positive trend. The preoperative stabilization of haemodynamics and improvement in organ function may contribute to favourable early postoperative outcomes.

Another advantage is the formation of myocardial scar tissue when the operation is delayed. Early intervention may lead to postoperative shunts due to the weak myocardium not being effectively held by sutures; conversely, performing the operation on fibrous myocardial scars is preferred by surgeons. Although Morimura *et al.* [17] did not objectively assess scar formation during the operation, they reported fibrous scar formation in a patient operated on 7 days after a myocardial infarction. In contrast, Coyan *et al.* [12] reported that despite delaying surgical intervention until 24 days after AMI onset, intraoperative findings showed visible scar formation; however, the infarcted tissue was still relatively fragile. In our study, patients who underwent an operation shortly after AMI onset naturally had an unclear VSR and fragile myocardium (Fig. 3a). However, for medium-term cases, despite the recognition of myocardial fragility, the location of the VSR could be clearly identified (Fig. 3b); when the period was longer, both scar formation and the VSR location were clearly visible (Fig. 3c and d). We also used long- and short-term treatment periods for reasons such as the potential for sudden haemodynamic collapse in PIVSR and the need for experienced surgeons due to myocardial fragility, allowing surgeons to schedule the operation at their convenience rather than as an emergency nighttime procedure.

However, prolonged support with MCS systems can lead to a higher incidence of thrombotic and haemorrhagic complications [24, 25]. In particular, haemorrhagic complications may be attributed to Impella use. First, the high shear forces and continuous flow of the Impella lead to the proteolysis of the high-molecular-weight von Willebrand factor, reducing platelet-binding affinity and causing acquired von Willebrand syndrome in a majority of patients within 24 h of initiating Impella use [26, 27]. The misplacement of an intracardiac device can aggravate this issue [28]. Second, the Impella’s distinctive purge system, which is designed to prevent blood from entering the motor, complicates anticoagulant therapy by impeding the absorption, deposition and clotting of blood components [27, 29]. Third, advancing liver failure further increases bleeding risk due to reduced levels of coagulation factors II, V, VII, IX and X [30]. Additionally, the simultaneous use of VA-ECMO or continuous haemodiafiltration may lead to increased platelet consumption [31]. In this study, despite RCC and PC transfusions, a significant decreasing trend in Hb and platelet counts was observed during the Impella treatment period. It was reported that 38.8% of the patients who received VA-ECMO exhibited severe thrombocytopaenia, compared with 76.9% of the patients with a low baseline platelet count [32]. Furthermore, among patients who received VA-ECMO support before VSR repair, 71.4% and 42.9% experienced major and fatal bleeding, respectively [14]. In our study, the most common device-related bleeding complications were nosebleeds (70%), followed by gastrointestinal bleeding and haematuria (30%) and cerebral haemorrhage (20%); no local wound infections were observed. Although no significant differences were found in the number of units of transfused RBCs, fresh-frozen plasma and PCs used during the Impella treatment period compared with the operation, it is important to acknowledge that our study may not have been adequately powered to detect small but clinically relevant differences. Therefore, the absence of significant differences should be interpreted with caution because the limited sample size and study design restrict the conclusiveness of these findings. Further research involving a larger cohort and more rigorous statistical analysis is necessary to definitively assess the impact of Impella and ECpella treatments on transfusion requirements.

This study has some limitations; first, its design as a small-cohort, single-centre, retrospective and observational study, combined with a brief follow-up period, restricts the breadth of its conclusions. Selection bias is unavoidable in this study, given its retrospective nature and the specific clinical setting, which may affect the generalizability of the results. The paucity of literature on advanced MCS for PIVSR highlights a significant gap in the evidence base, and with only 10 participants, our study’s findings are difficult to compare with those of larger studies. Furthermore, the absence of a comparator for the Impella precludes attributing the observed outcomes exclusively to its use. Therefore, to accurately assess the effectiveness of the Impella as a treatment strategy for PIVSR, future studies with larger sample sizes, comparative analyses with alternative interventions and extended follow-up periods are warranted.

## CONCLUSION

In patients with PIVSR complicated by CS, preoperative treatment with the Impella or ECpella may improve outcomes by stabilizing the patient’s haemodynamics and facilitating organ failure recovery before the operation. However, the drawbacks of prolonged support include a high incidence of haemorrhagic complications, extended mechanical ventilation and transfer for rehabilitation. Further investigations are necessary to determine the optimal duration of support, particularly considering these issues.

## Data Availability

The data underlying this article will be shared upon reasonable request addressed to the corresponding author.

## Notes

An abstract of this manuscript has not been submitted at the EACTS or ESTS annual meetings.

## ABBREVIATIONS

AMI: Acute myocardial infarction
CI: Cardiac index
CK: Creatine kinase
CS: Cardiogenic shock
Hb: Haemoglobin
HF: Heart failure
IABP: Intra-aortic balloon pump
IQR: Interquartile range
MCS: Mechanical circulatory support
PC: Platelet concentrate
PIVSR: Postinfarction ventricular septal rupture
RBC: Red cell concentrate
VA-ECMO: Veno-arterial extracorporeal membrane oxygenation
VSR: Ventricular septal rupture
